# Altered inflammasome machinery as a key player in the perpetuation of Rett syndrome oxinflammation

**DOI:** 10.1016/j.redox.2019.101334

**Published:** 2019-10-06

**Authors:** Alessandra Pecorelli, Valeria Cordone, Nicolò Messano, Changqing Zhang, Stefano Falone, Fernanda Amicarelli, Joussef Hayek, Giuseppe Valacchi

**Affiliations:** a Plants for Human Health Institute, Dept. of Animal Science, NC Research Campus, NC State University, Kannapolis, 28081, NC, USA; b Dept. of Biomedical and Specialist Surgical Sciences, University of Ferrara, 44121, Ferrara, Italy; c Plants for Human Health Institute, Dept. of Plant and Microbial Biology, NC Research Campus, NC State University, Kannapolis, 28081, NC, USA; d Dept. of Life, Health and Environmental Sciences, University of L'Aquila, 67100, L'Aquila, Italy; e Child Neuropsychiatry Unit, University General Hospital, Azienda Ospedaliera Universitaria Senese, 53100, Siena, Italy; f Dept. of Food and Nutrition, Kyung Hee University, 02447, Seoul, South Korea

**Keywords:** NLRP3, ASC, Inflammatory status, Cytokines, MeCP2

## Abstract

Rett syndrome (RTT) is a progressive neurodevelopmental disorder mainly caused by mutations in the X-linked MECP2 gene. RTT patients show multisystem disturbances associated with an oxinflammatory status. Inflammasomes are multi-protein complexes, responsible for host immune responses against pathogen infections and redox-related cellular stress. Assembly of NLRP3/ASC inflammasome triggers pro-caspase-1 activation, thus, resulting in IL-1β and IL-18 maturation. However, an aberrant activation of inflammasome system has been implicated in several human diseases. Our aim was to investigate the possible role of inflammasome in the chronic subclinical inflammatory condition typical of RTT, by analyzing this complex in basal and lipopolysaccharide (LPS)+ATP-stimulated primary fibroblasts, as well as in serum from RTT patients and healthy volunteers. RTT cells showed increased levels of nuclear p65 and ASC proteins, pro-IL-1β mRNA, and NLRP3/ASC interaction in basal condition, without any further response upon the LPS + ATP *stimuli*. Moreover, augmented levels of circulating ASC and IL-18 proteins were found in serum of RTT patients, which are likely able to amplify the inflammatory response. Taken together, our findings suggest that RTT patients exhibited a challenged inflammasome machinery at cellular and systemic level, which may contribute to the subclinical inflammatory state feedback observed in this pathology.

## Highlights

• RTT cell shows a constitutive NFκB activation.
• Aberrant activation of inflammasome system is evident in RTT.
• This new evidence can explain the demonstrated subclinical inflammation in RTT.

## Graphical abstract

**Figure:**
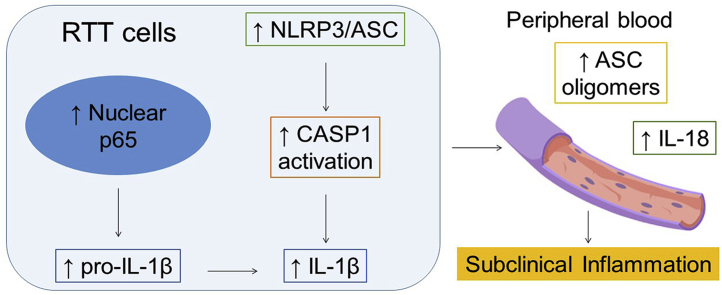

## 1 Introduction

Rett syndrome (RTT; OMIM identifier #312750), first described by the physician Andreas Rett [1], is a severe neurodevelopmental disorder, predominantly affecting females (approx. 1 per 10,000 live births) [2]. In 90–95% of patients diagnosed with RTT, the disorder is due to *de novo* loss-of-function mutations in the X-linked *MECP2* gene, which encodes methyl-CpG binding protein 2 [3].

After 6-18-months of apparently normal development [4], RTT leads to both developmental regression, involving loss of hand skills, motor skills and speech, and a number of co-morbidities, including breathing disturbances, seizures, gastro-intestinal complications, and scoliosis [5], allowing to define RTT as a ‘spectrum disorder’. Today, the complete pathogenic mechanisms linking MECP2 dysfunction to RTT symptoms are still not clear.

In the last decade, a growing body of evidence supported the idea that an OxInflammation condition, observed in brain and peripheral compartments of both RTT patients and animal models, could be involved in RTT pathophysiology [6]. This condition in RTT is due to the concomitant dysregulation of redox and immune homeostasis and characterized by a subclinical inflammatory status coupled with an increased production of oxidant species and a perturbed defense response [[6], [7], [8], [9]]. Based on recent findings, cytosolic molecular complexes termed inflammasomes have emerged as central mediators in the crosstalk between redox imbalance and inflammation associated with a wide range of diseases [10]. The assembly of inflammasomes occurs following the recognition of multiple diverse endogenous and exogenous signals such as “pathogen-associated” or “danger-associated molecular patterns” (PAMPs or DAMPs) by a cytosolic subset of “pattern recognition receptors” (PRRs), named “nucleotide-binding domain and leucine-rich repeat-containing (NLR) proteins” [11]. The activation of these intracellular sensors such as NLRP3, the most widely studied member among them, triggers the oligomerization and the recruitment of the adaptor protein ASC, which in turn is able to recruit the effector protein pro-caspase 1 [12]. The proximity-induced pro-caspase 1 auto-cleavage leads to the generation of the catalytically active caspase 1 (CASP1), which prompts the downstream responses, consisting of the conversion of both pro-IL-1β and pro-IL-18 to their biologically active forms, and/or the proteolytic action of gasdermin D (GSDMD). This latter promotes the release of GSDMD N-terminal fragments that oligomerize into ring-shaped structures in membranes. GSDMD pores allow cells to release passively their cytoplasmic content in a size-dependent manner (*i.e.,* cytokines like IL-1β) [13] or drive cells towards pyroptotic death [11]. Besides its involvement in innate immune responses, NLRP3 inflammasome has been demonstrated to have a role in a wide range of inflammatory diseases, cancer, metabolic and autoimmune disorders, and aging [[14], [15], [16], [17], [18], [19]]. The inflammasome pathway is implicated even in the neuroinflammation observed in neurodegenerative disorders, like multiple sclerosis, Alzheimer's and Parkinson's disease [20,21] and in neurodevelopmental pathologies, like autistic spectrum disorders (ASDs) [22].

Since the inflammasome pathway could contribute to the characteristic OxInflammatory status of RTT, this work was aimed at investigating the activation state of the inflammasome in fibroblasts and serum samples from RTT patients. We revealed that a deregulated activation of inflammasome pathway occurs in RTT, as proved by the increased constitutive levels of inflammasome components in patient-derived serum and fibroblast cell lines and by the inability of RTT cells to activate properly this pathway after further pro-inflammatory challenges.

## 2 Materials and methods

### 2.1 Antibodies

Cell Signaling Technology, Inc. (Danvers, MA, USA) supplied the following antibodies: anti-nuclear factor κ-light-chain-enhancer of activated B cells (NF-κB) p65 subunit antibody (cat. 8242; dil. 1:1,000); anti-NACHT, LRR and PYD domains-containing protein 3 (NLRP3) antibody (cat. 13158; dil. for WB 1:500 and for IF 1:100); anti-caspase 1 (CASP1) antibody (cat. 2225; dil. 1:1,000), anti-cleaved-IL-1β (cat. 83186; dil. 1:100); anti-glyceraldehyde-3-phosphate dehydrogenase (GAPDH) (cat. 5174; dil. 1:5,000); anti-histone deacetylase 1 (HDAC1) antibody (cat. 8242; dil. 1:1,000), and peroxidase-conjugated anti-rabbit secondary antibody (cat. 7074; dil. 1:10,000). Santa Cruz Biotechnology, Inc. (Santa Cruz, CA) provided the anti-apoptosis-associated speck-like protein containing CARD (ASC) (cat. sc-271054; dil. for WB 1:800 and for IF 1:200) antibody. Both AlexaFluorTM 488 goat anti-rabbit IgG (H + L) (cat. A-11008; dil. 1:1,000) and AlexaFluorTM 568 goat anti-mouse IgG (H + L) (cat. A-11004; dil. 1:1,000) antibodies were provided by Thermo Fisher Scientific (Waltham, MA, USA). The peroxidase-conjugated anti-mouse secondary antibody (cat. 170–6515; dil. 1:10,000) was supplied by Bio-Rad Laboratories (Hercules, CA, USA).

### 2.2 Study approval

Female patients with classical RTT and healthy sex- and age-matched controls were enrolled for the study. All patients were admitted to the Child Neuropsychiatry Unit of the University Hospital of Siena (Siena, Italy). Diagnosis and inclusion/exclusion criteria of RTT were set in agreement with revised RTT nomenclature consensus [4]. The study was conducted in accordance with the Code of Ethics of the World Medical Association (Declaration of Helsinki), and the protocol was approved by the Ethics Committee of Institutional Review Board of University Hospital, Azienda Ospedaliera Universitaria Senese (AOUS), Siena, Italy. A written form of the informed consents was signed from either the parents or the legal tutors of the participants.

### 2.3 Human fibroblasts culture

Skin biopsies from healthy donors (CTR; N = 6) were taken during health checks or by donations, while skin biopsies from RTT patients (N = 6; age: 20 ± 3.8, expressed as mean ± SD) were obtained during the periodic clinical checks-up. Human skin fibroblasts were isolated by 3-mm skin punch biopsy, as previously described [23]. Before the experimental procedure, fibroblasts were stained for Vimentin and checked for mycoplasma contamination. Cells were cultured with DMEM medium, containing 10% (v/v) fetal bovine serum (cat. 10-014-CV and cat. 35-011-CV, respectively, Corning, New York, NY, USA), antibiotics (100 IU/ml penicillin, 100 mg/ml streptomycin) (cat. 30-002-CI; Corning) and incubated in humidified atmosphere (5% CO_2_) at 37 °C. All experiments were performed by using fibroblasts between the third and fifth passage *in vitro*.

### 2.4 Cell treatment with LPS and ATP

CTR and RTT fibroblasts were seeded (5,000 cells/cm^2^) in complete medium and after 48 h, cells were starved with 1% FBS-containing medium for 15 h to minimize cell proliferation. Cells were then incubated with or without 100 μg/ml lipopolysaccharide (LPS, dissolved in water), for 6 h in 1% FBS-supplemented medium and with 5 mM adenosine triphosphate (ATP, dissolved in water) for additional 30 min (cat. L2630 and cat. A6419, respectively, Sigma-Aldrich, St. Louis, MO, USA). The LPS concentration was calculated based on the evaluation of *NLRP3* gene expression, in response to different concentrations (0, 10, 50, 100 and 200 μg/ml for 6 h), while the ATP treatment was established based on literature papers [24,25]. The chosen concentration of LPS (100 μg/ml) induced an inflammasome-related response and no cell death was observed (Supplementary Fig. S1).

### 2.5 Western immunoblot analysis

Fibroblasts were lysed in RIPA buffer (cat. J62524, Alfa Aesar, Tewksbury, MA, USA), supplemented with 1% (v/v) protease inhibitors and 1% (v/v) phosphatase inhibitors (cat. 78430 and cat. 1862495, respectively, Thermo Fisher Scientific, Waltham, MA, USA). After three freezing-thawing cycles, cell lysates were centrifuged at 17,000×*g* for 15 min at 4 °C, and supernatants were used for the evaluation of total protein concentration, by using the Quick Start™ Bradford Protein Assay Kit (cat. 5000201, Bio-rad Laboratories) and bovine serum albumin (BSA) as standard. Samples were denatured and run in triplicates on 10–15% polyacrylamide gels, as previously reported [26]. Proteins were transferred from polyacrylamide gels onto polyvinylidene difluoride (PVDF) membranes by electrophoretic transfer. Non-specific binding sites were blocked at room temperature for 1 h with 5% (w/v) Blotting-Grade Blocker (cat. 170–6404, Bio-Rad Laboratories), in Tris-buffer saline containing 0.1% (v/v) Tween-20 (cat. P5927, Sigma–Aldrich) (TBS-T). Membranes were first incubated overnight with primary antibodies-containing TBS-T (see the antibodies section), and then with anti-rabbit or anti-mouse peroxidase-conjugated secondary antibodies diluted in TBS-T for 2 h at room temperature (see the antibodies section). The protein bands were detected by using Clarity™ Western ECL Substrate Kit (cat. 1705060, Bio-Rad Laboratories) and ChemiDoc™ MP Imaging System hardware and software (Bio-Rad Laboratories). Images of bands were analyzed by the Nonlinear Dynamics TotalLab software (TotalLab Ltd, Newcastle upon Tyne, UK). Data were normalized against HDAC1 or GAPDH, depending on nuclear or cytosolic/total proteins analyzed, and results were given as arbitrary units.

### 2.6 Subcellular protein fractionation

Nuclear and cytoplasmic protein fractions were prepared by using the Nuclear Extraction Kit (cat. 2900, Merck Millipore, Burlington, MA, USA). Briefly, cells were washed with PBS and then were lysed with Cytoplasmic Lysis Buffer, containing 0.5 mM dithiothreitol (DTT) and 0.1% (v/v) protease inhibitor cocktail. After homogenization, the disrupted cell suspension was centrifuged at 8,000×*g* for 20 min at 4 °C, and the supernatant, containing the cytosolic portion, was recovered. The remaining pellet was then resuspended in Nuclear Extraction Buffer supplemented with 0.5 mM DTT and 0.1% (v/v) protease inhibitor cocktail and homogenized. The resulting nuclear suspension was centrifuged at 16,000×*g* for 5 min at 4 °C and the supernatant, containing the nuclear extract, was collected [27]. Total protein concentration was determined by Bradford analysis (Quick Start™ Bradford Protein Assay Kit, cat. 5000201, Bio-rad Laboratories).

### 2.7 RNA extraction and real time RT-PCR analysis

As previously reported [28], total RNA was extracted from fibroblasts by using Aurum Total RNA Mini Kit (cat.732–6820, Bio-Rad), removing genomic contamination by using DNase I, as recommended by the supplier. RNA (1 μg) was converted into complementary DNA by using iScript Reverse Transcription kit (cat. 1708841, Bio-Rad). The obtained cDNA (diluted 1:10) was used for the real time PCR step with SsoAdvanced Universal SYBR Green Supermix (cat. 172–5271, Bioline, London, UK) in a LightCycler® 480 Instrument (Roche, Indianapolis, IN, USA). Primers were synthetized by Sigma-Aldrich (St. Louis, MO, USA): *NLRP3* (forward, 5′-CGGGGCCTCTTTTCAGTTCT-3′; reverse, 5′-CCCCAACCACAATCTCCGAA-3′) (Primer BLAST, accession number: NM_004895.4); *CASP1* (forward, 5′-CCGTTCCATGGGTGAAGGTA-3′; reverse, 5′-TGCCCCTTTCGGAATAACGG-3′) (Primer BLAST, accession number: NM_033292.4); *IL-1β* (forward, 5′-CACGATGCACCTGTACGATCA-3'; reverse, 5′-GTTGCTCCATATCCTGTCCCT-3′) [58]; *GAPDH* (forward, 5′-TGACGCTGGGGCTGGCATTG-3′; reverse, 5′- GGCTGGTGGTCCAGGGGTCT-3′) [26].

The PCR protocol was set as follows: polymerase activation and initial denaturation at 95 °C for 30 s, and 40 cycles of 95 °C for 15 s and 60 °C for 60 s. In order to check the presence of possible co-amplified undesired targets, melt-curve analysis was performed for all primer pairs (65 °C–95 °C, 0.5 °C increment, 2 s/step). Quantitative relative gene expression was calculated by using the 2^−ΔΔCt^ method [29], using GAPDH as the reference mRNA and one of the controls as the internal calibrator. Each sample was processed by analyzing three replicates.

### 2.8 Immunofluorescence analysis

Fibroblasts were seeded (5,000 cells/cm^2^) and grown on coverslips in complete medium. CTR and RTT cells were starved and treated with LPS + ATP, as described in the “Cell treatment with LPS and ATP” sub-section. As previously reported [23], cells were washed twice with d-phosphate buffered saline (PBS), then fixed in 10% (w/v) neutral buffered formalin solution for 10 min, and permeabilized in PBS containing 0.25% (v/v) Triton X-100 for 10 min at 4 °C. Non-specific binding sites were blocked with 3% (w/v) BSA in PBS for 30 min. Cells were then incubated with primary antibodies that were diluted in PBS containing 0.5% (w/v) BSA at 4 °C overnight (see the “Antibodies” section for information about dilutions). After three washes with PBS, cells were incubated with AlexaFluor 488 or AlexaFluor 568 antibodies-containing PBS at 4 °C for 1 h (see the “Antibodies” section for information about dilutions). After five washes with PBS, cells were incubated with a solution of 4′,6-diamidino-2-phenylindole (DAPI)-containing PBS for 1 min (dil. 1:20,000) and mounted with ProLong Diamond mounting medium (cat. D1306 and cat. P36965, respectively, Thermo Fisher Scientific). Cells were observed and photographed by confocal microscopy (objective 40×) by using a Zeiss LSM 710 microscope (Carl Zeiss, Thornwood, NY, USA) and Zen 2008 Software (Carl Zeiss Microscopy GmbH, Jena, Germany). Digital images were analyzed by using an open source Java-based Fiji-ImageJ image processing package, and the coloc2 plug-in for colocalization. Results were given as Pearson coefficients [30].

### 2.9 Serum sampling

Serum samples were obtained from female patients with clinical diagnosis of typical RTT and MECP2 mutation (n = 42; median age: 15) and healthy controls (n = 16; median age: 16). As previously reported [31], fasting venous blood was collected at 8–10 a.m. following an overnight fast and all manipulations were carried out within 2 h. Blood was collected in tubes without anticoagulants and allowed to clot at RT. Following centrifugation at 1,500×*g* for 10 min, the sera were transferred into clean tubes. Serum samples were stored at −80 °C for further immunoblot (ASC and CASP1) or ELISA (IL-18) analyses.

### 2.10 ASC oligomerization assay

For the ASC oligomerization assay, five μl of serum were suspended in 500 μL PBS and cross-linked with 2 mM disuccinimidyl suberate (DSS) for 30 min a room temperature. Cross-linked samples were centrifuged at 10,000×*g* for 15 min at 4 °C. The pellets containing ASC oligomers were boiled with 2x protein loading buffer for 5 min for Western blot analysis.

### 2.11 Enzyme-linked immunosorbent assay (ELISA)

Serum concentrations of IL-18 were determined by ELISA using a commercial kit (RayBiotech Life, Peachtree Corners, GA, USA), according to the manufacturer's instructions. All samples were analyzed in duplicate. A calibration curve was performed using IL-18 as a standard. The optical absorbance was measured with a microplate reader at 450 nm, and results are expressed as pg/mL. The lower limit of detection for IL-18 was 0.5 pg/mL.

### 2.12 Statistics

Statistical analyses were performed by using GraphPad Prism 6 and Statsoft Statistica10 softwares. One-way or factorial ANOVA, with *post-hoc* Tukey's tests were applied. The null hypothesis was rejected with P less than 0.05. All data were expressed as means ± standard deviations (SD).

## 3 Results

### 3.1 Constitutive nuclear translocation of NF-κB p65 and increased levels of cleaved IL-1β in RTT fibroblasts

**Fig. 1:**
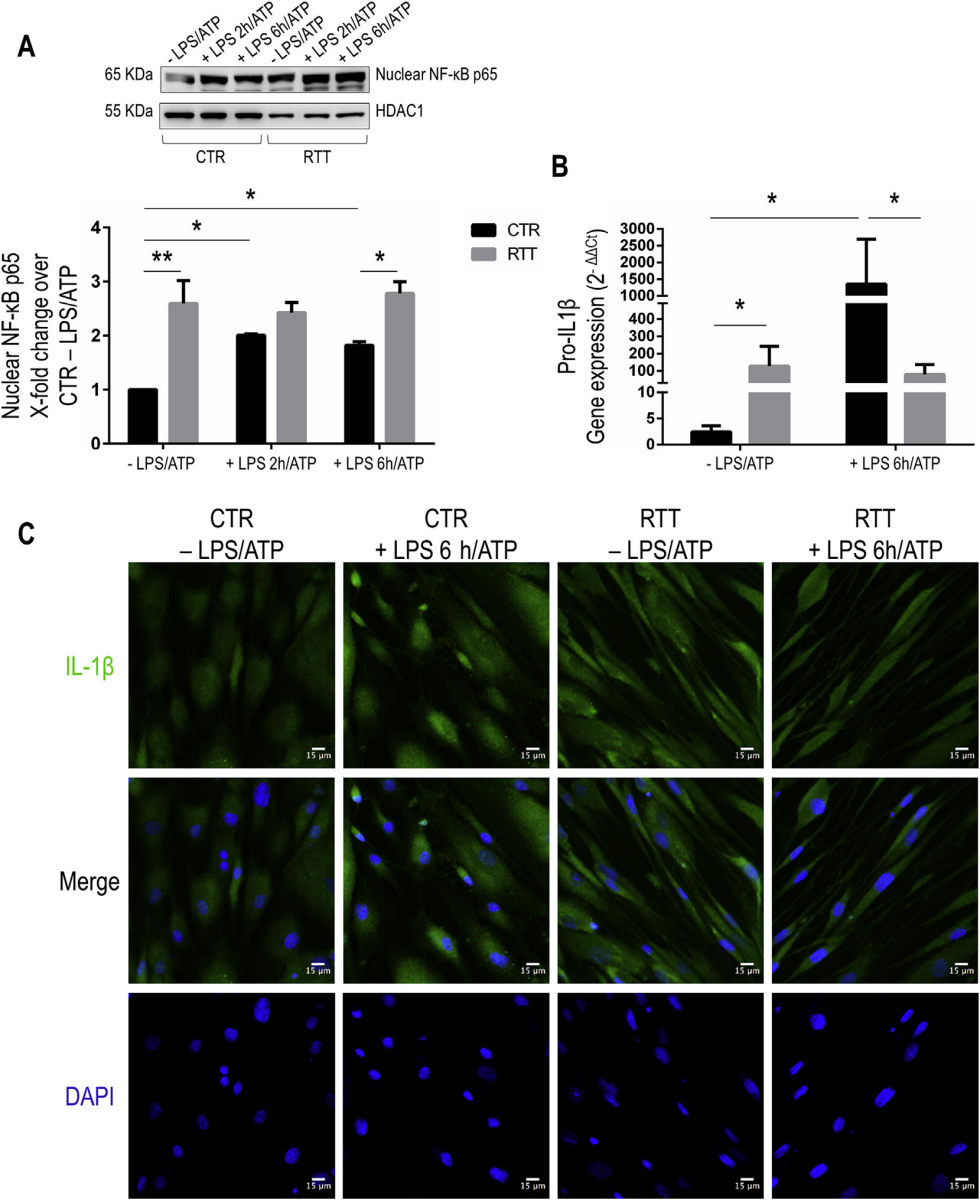
Constitutive nuclear translocation of NF-κB p65 and expression of cleaved IL-1β in RTT fibroblasts. **Panel A.** Representative Western blots for NF-κB p65 in nuclear extracts from control and RTT fibroblasts stimulated with LPS 100 μg/ml for 2 and 6 h plus ATP 5 mM for 30 min. Quantification is showed in bottom panel. Data are given as means ± SD. **Panel B.** The mRNA levels of pro-IL-1β were analyzed by real-time RT-PCR. Data are expressed as 2^−ΔΔCt^, using GAPDH as the reference, and one of the controls as the internal calibrator. Data are given as means ± SD. **Panel C.** Immunofluorescence for cleaved IL-1β in control and RTT fibroblasts stimulated with LPS 100 μg/ml for 6 h and ATP 5 mM for 30 min. Nuclei are stained with DAPI. Bar = 15 μm. CTR, control; RTT, Rett syndrome; LPS, lipopolysaccharide; ATP, adenosine triphosphate. *P < 0.05; **P < 0.01. Results were analyzed by factorial ANOVA (with 2 × 2 × 3 design for panel A, and 2 × 2 design for panel B), with post-hoc Tukey's multiple comparisons test.

First critical step in inflammasome activation is the transcriptional up-regulation of inflammasome components via nuclear factor (NF)-κB signaling. As shown in Fig. 1A, RTT fibroblasts revealed highly augmented levels of nuclear NF-κB p65 subunit in basal condition (P < 0.01), as compared to unstimulated CTR. However, while CTR fibroblasts responded to LPS + ATP treatment by increasing p65 nuclear translocation over time (after 2 and 6 h) (P < 0.05), p65 nuclear levels did not increase any further after LPS + ATP in RTT fibroblasts (Fig. 1A).

NF-κB–activating *stimulus* induces elevated expression of pro-IL-1β, a key pro-inflammatory cytokine involved in the inflammasome pathway [32]. RTT fibroblasts displayed a robust constitutive pro-IL-1β mRNA expression in basal condition when compared to unstimulated CTR cells (P < 0.05) (Fig. 1B). As expected, increased transcriptional levels of pro-IL-1β have been detected in LPS + ATP-stimulated CTR cells (P < 0.05; as compared to untreated CTR); whereas no further changes in pro-IL-1β levels were noticed in RTT fibroblasts after the pro-inflammatory stimulation (Fig. 1B).

In line with these results, immunofluorescence analysis for the cleaved IL-1β revealed an increase of green signal in cytoplasmic compartment of untreated RTT cells as compared to basal CTR fibroblasts (Fig. 1C). No change in fluorescence intensity was observed in RTT fibroblasts upon LPS + ATP stimulation when compared to the unstimulated condition (Fig. 1C). On the other hand, an increased green fluorescence for the mature IL-1β was evident in CTR fibroblasts after LPS + ATP treatment (Fig. 1C).

### 3.2 NLRP3 inflammasome components are constitutively expressed at high levels in RTT fibroblasts

**Fig. 2:**
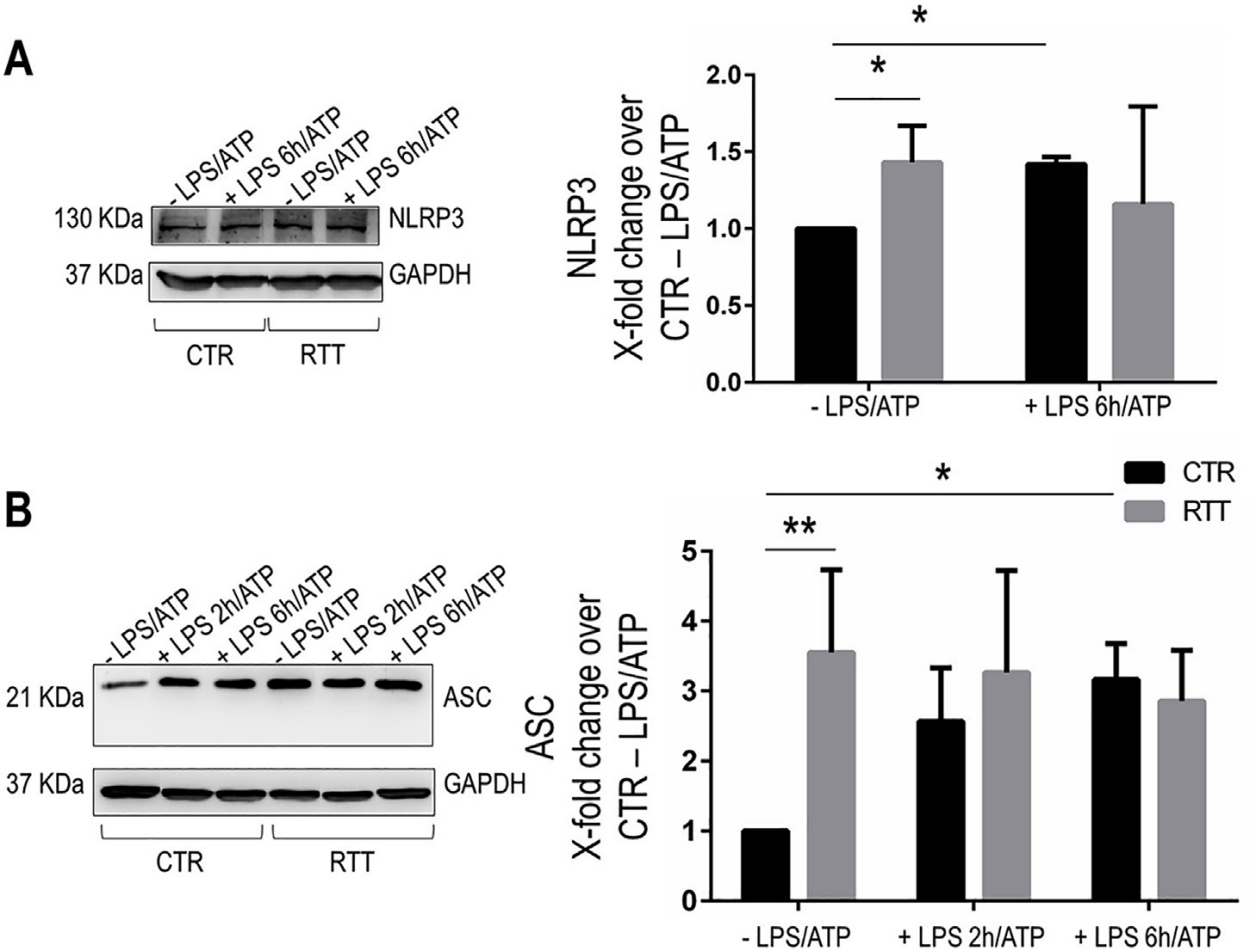
Increased levels of NLRP3 and ASC in basal RTT fibroblasts. **Panel A.** Representative images and densitometric analysis of Western blotting for NLRP3 in control and RTT fibroblasts incubated without or with LPS 100 μg/ml for 6 h plus ATP 5 mM. **Panel B.** Representative images and densitometric analysis of Western blots for ASC in control and RTT fibroblasts stimulated with LPS 100 μg/ml for 2 and 6 h plus ATP 5 mM. Data are given as means ± SD. CTR, control; RTT, Rett syndrome; LPS, lipopolysaccharide; ATP, adenosine triphosphate. *P < 0.05; **P < 0.01. Results were analyzed by Two-way ANOVA, with post-hoc Tukey's multiple comparisons test.

In addition to NF-κB p65, other key players in the inflammasome system were analyzed, including NLRP3 and ASC. Basal RTT fibroblasts showed higher NLRP3 protein levels compared to CTR (P < 0.05), without any evident change after pro-inflammatory stimulation (Fig. 2A). Instead, NLRP3 levels in CTR fibroblasts were increased significantly upon LPS + ATP treatment (P < 0.05; as compared to untreated CTR) (Fig. 2A).

Similarly, an increase of ASC levels could be detected in basal RTT fibroblasts as compared to untreated CTR (P < 0.01), but with no statistically significant variation in response to the pro-inflammatory *stimuli* (Fig. 2B). On the other hand, in CTR cells ASC levels were low in basal conditions, but significantly increased after LPS + ATP stimulation (P < 0.05) (Fig. 2B).

### 3.3 RTT fibroblasts show an increased colocalization between NLRP3 and ASC

**Fig. 3:**
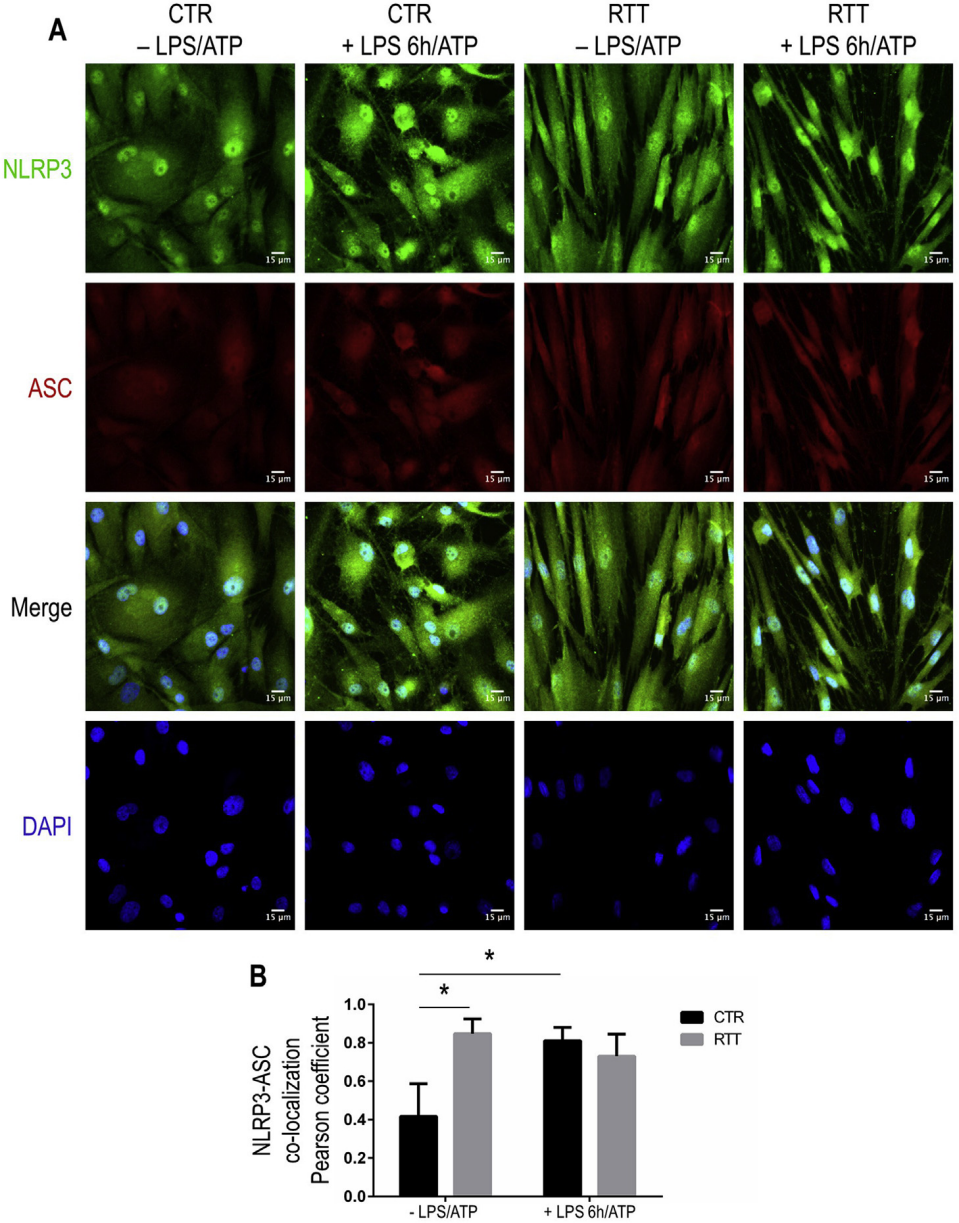
Increased formation of NLRP3/ASC inflammasome complexes in RTT fibroblasts. **Panel A.** Confocal images showing localization of NLRP3 (green fluorescence) and ASC (red fluorescence) in control and RTT fibroblasts stimulated with LPS 100 μg/ml for 6 h and ATP 5 mM for 30 min. Overlay of the green and red channel shows that there is colocalization between NLRP3 and ASC. Nuclei are stained with DAPI. Bar = 15 μm. CTR, control; RTT, Rett syndrome; LPS, lipopolysaccharide; ATP, adenosine triphosphate. *P < 0.05. **Panel B.** Pearson's correlation coefficient values for colocalization of NLRP3 and ASC. Co-localization data were given as means ± SD. Results were analyzed by Two-way ANOVA, with post-hoc Tukey's multiple comparisons test. (For interpretation of the references to colour in this figure legend, the reader is referred to the Web version of this article.)

To determine if high constitutive levels of the inflammasome components in RTT cells paralleled with inflammasome assembly, we assessed the NLRP3-ASC interaction by evaluating their cellular localization by immunofluorescence (Fig. 3).

In CTR cells, the LPS + ATP treatment induced an increase of the NLRP3-related immunofluorescence signal into the cytoplasmic and perinuclear region (likely, endoplasmic reticulum), as well as an enhanced ASC-related signal into the cytosol, as compared to unstimulated CTR cells (Fig. 3A). Conversely, in RTT fibroblasts, basal signals of both NLRP3 and ASC are higher and localized in the cytosolic compartment, whereas LPS + ATP stimulation did not affect the cytosolic localization (Fig. 3A).

After co-localization analysis, we found that a significant NLRP3-ASC interaction was induced by LPS + ATP only in CTR fibroblasts (P < 0.05); while, RTT cells showed a significant increase of inflammasome assembly already in basal condition (P < 0.05; as compared to untreated CTR), and were unable to respond to further challenge with LPS + ATP (Fig. 3B).

### 3.4 Altered levels of pro-CASP1 and active CASP1 form in RTT fibroblasts

The key outcome of NLRP3-ASC assembly is the self-cleavage and activation of pro-CASP1 that, then, leads to the processing and maturation of the pro-inflammatory cytokines IL-1β and IL-18 [12]. Given the higher levels of cleaved IL-1β and inflammasome assembly in RTT cells, we next evaluated the protein expression of the pro-form and active form of CASP1.

**Fig. 4:**
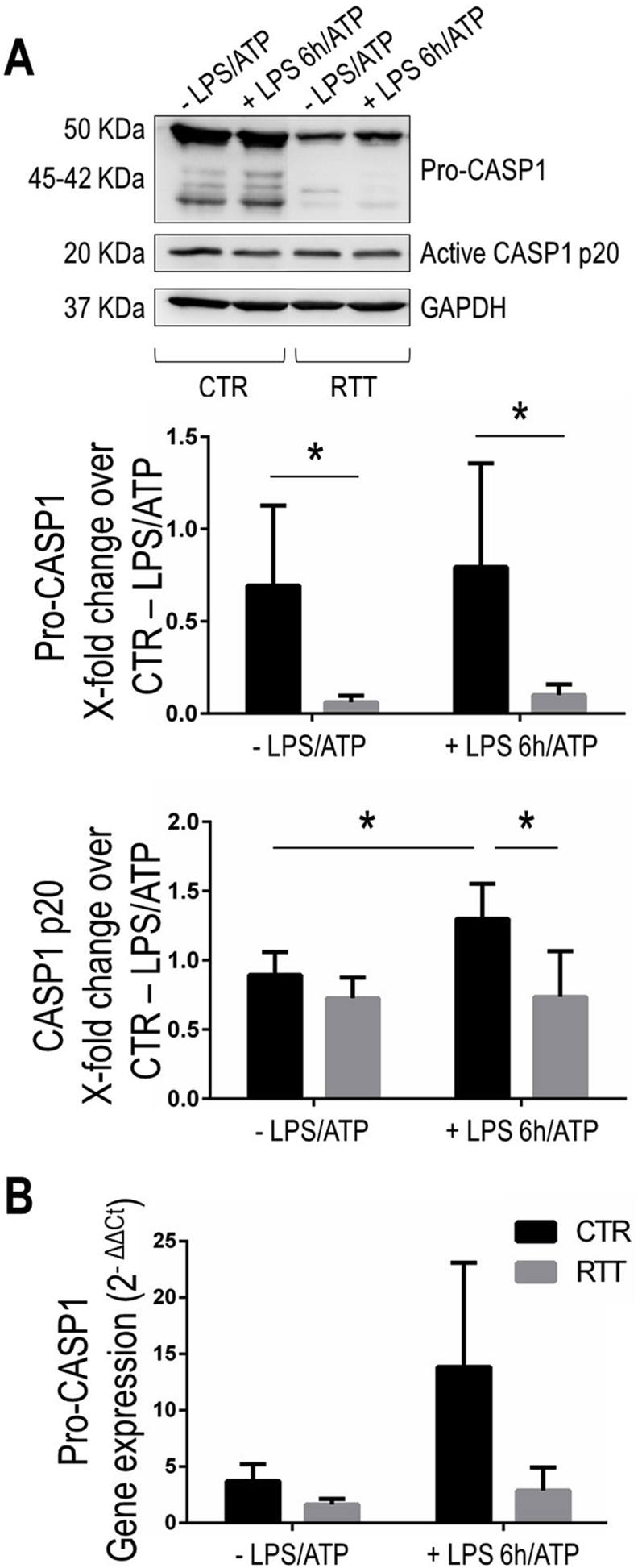
Altered protein and mRNA levels of caspase 1 in RTT fibroblasts. **Panel A.** Representative Western blots images for pro-CASP1 and active CASP1 p20 in control and RTT fibroblasts stimulated with LPS 100 μg/ml for 6 h and ATP 5 mM for 30 min. Quantification is showed in bottom panel. Data were given as means ± SD. CTR, control; RTT, Rett syndrome; LPS, lipopolysaccharide; ATP, adenosine triphosphate. *P < 0.05. Results were analyzed by Two-way ANOVA, with post-hoc Tukey's multiple comparisons test. **Panel B.** The mRNA levels of pro-CASP1 were analyzed by real-time RT-PCR. Data are expressed as 2^−ΔΔCt^, using GAPDH as the reference, and one of the controls as the internal calibrator. Data are given as means ± SD. CTR, control; RTT, Rett syndrome; LPS, lipopolysaccharide; ATP, adenosine triphosphate. *P < 0.05. Results were analyzed by Two-way ANOVA, with post-hoc Tukey's multiple comparisons test.

In both basal and LPS + ATP stimulated conditions, RTT fibroblasts displayed low pro-CASP1 levels respect to the CTR cells (P < 0.05) (Fig. 4A). The basal levels of CASP1 p20 active form were similar between CTR and RTT cells, while the treatment with LPS + ATP increased its levels in CTR cells (P < 0.05), no change was observed in RTT fibroblasts after the pro-inflammatory stimulus (Fig. 4A).

To verify if regulation of pro-CASP1 protein expression occurs at the transcription level, we assessed its mRNA expression by real time PCR. As showed in Fig. 4B, in unstimulated conditions, RTT cells displayed a trend in reduction of pro-CASP1 mRNA levels compared to CTR fibroblasts. After LPS + ATP stimulation, pro-CASP1 gene expression showed a trend in increase in CTR cells, while no significant changes were observed in RTT fibroblasts (Fig. 4B).

### 3.5 Increased release of inflammasome components in serum of RTT patients

**Fig. 5:**
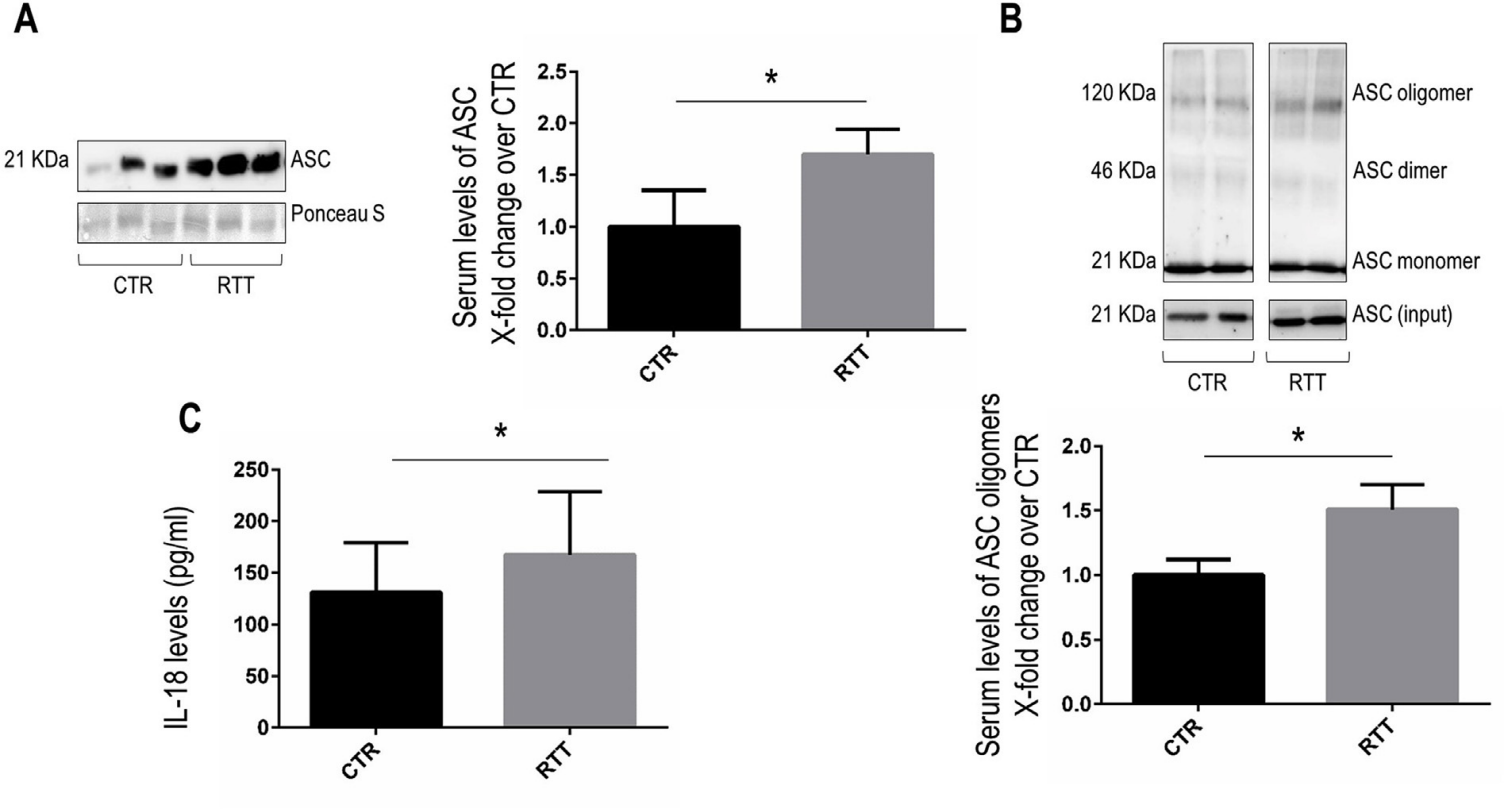
Increased release of inflammasome components in serum of RTT patients. **Panel A.** Representative Western blots images for ASC in serum samples from control subjects and RTT patients. Quantification is showed in right panel. **Panel B.** Representative images and densitometric analysis of Western blotting for ASC oligomers in serum samples from control subjects and RTT patients. Quantification is showed in bottom panel. **Panel C.** Serum levels of IL-18 in control subjects and RTT patients were quantified by ELISA. For all the panels, the results are presented as means ± SD. CTR, control; RTT, Rett syndrome; *P < 0.05. Results were analyzed by *t*-test for independent groups (CTR N = 17; RTT N = 41).

A growing body of evidence supports a model whereby the cellular release of inflammasome proteins through extracellular vesicles such as exosomes delivers their cargo *in vivo* to amplify the inflammatory signaling in peripheral tissues [33,34]. Therefore, to further study inflammasome activation in RTT, we evaluated the protein expression of the main inflammasome components in serum from RTT patients and control subjects. Consistent with the results obtained in fibroblasts, we found that ASC protein levels were higher in the serum of RTT patients than in the control group (Fig. 5A). Furthermore, immunoblot analysis of DSS-crosslinked ASC oligomers further confirmed a significant increase of catalytically active ASC oligomeric species in RTT serum samples (Fig. 5B). Next, as hallmark of inflammasome activation, we measured the serum levels of IL-18 by ELISA. As showed in Fig. 5C, serum IL-18 concentrations were significantly higher in RTT patients than in control subjects (p < 0.05).

## 4 Discussion

The aim of our work was to elucidate the possible involvement of the inflammasome pathway as a key player in RTT subclinical inflammation. Inflammasomes, cytoplasmic multi-protein complexes, provide host immune defense against a diverse range of pathogen infections and cellular stress signals [11]. As previously mentioned, assembly of NLRP3/ASC inflammasome leads to subsequent recruitment and autocatalytic activation of CASP1 that, finally, promotes the maturation of IL-1β and IL-18 [11,35]. These key pro-inflammatory cytokines are able to promote a multitude of finely regulated immune responses useful for restoring the physiological conditions [11]. However, it is also well known that an uncontrolled and prolonged inflammasome activation with an excessive release of cytokines can participate in the onset and progression of chronic inflammatory states associated with a wide variety of human diseases [10,36,37].

Although RTT is primarily a genetic brain disorder with prominent neurological symptoms, a vast body of evidence supports a key role for the interplay between redox imbalance and a subclinical inflammatory status (oxinflammation phenomenon) in different systemic abnormalities observed in RTT patients (*i.e.,* mitochondrial dysfunctions, metabolic alterations, immune dysregulation, gastrointestinal problems, breathing disturbances and recurrent infections) [6,38]. In this contest, the dysregulation of NLRP3 inflammasome pathway could be a new molecular mechanism able to contribute to RTT pathophysiology, in both brain and periphery.

In general, although still under debate [39], it is accepted that the molecular mechanisms leading to the inflammasome activation are mainly driven by two different signals. Typically, ‘signal 1’ or ‘priming’ molecules (*e.g.*, LPS) induce the expression of NLRP3, pro-IL-1β and pro-IL-18 *via* NF-κB activation [32,39]. Moreover, PAMPs or DAMPs (*e.g.,* ATP, particulate matter, heme, pathogen-associated RNA, etc) activate the NLRP3 inflammasome assembly (‘signal 2’) [32]. The particular interest towards NLRP3 inflammasome lies in the broad range of molecular and cellular signaling events that are induced by its activators among which there are ionic flux (*e.g.,* K^+^, Na^+^, chloride fluxes and Ca^2+^ signaling), ROS production, lysosomal destabilization, mitochondrial dysfunction and post-translational modifications of NLRP3 [40,41]. Of note, some of these stimuli are present in RTT patients [42,43] and, therefore, could promote a dysregulated NLRP3 inflammasome function that, in turn, could contribute to the typical RTT subclinical inflammation [6,38,44].

In the present work, RTT cells showed an increased steady level of nuclear NF-κB p65 subunit, as compared to CTR cells. A significant increase of NF-κB p65 nuclear translocation was found in CTR cells after LPS + ATP treatment, whereas RTT fibroblasts did not undergo any significant change in the transcription factor nuclear levels in response to the inflammatory *stimuli*. These peculiar results suggest that, in basal conditions, RTT fibroblasts exhibit a marked NF-κB p65 activation which amplitude cannot be achieved by control cells even upon pro-inflammatory stimulation. This indicates the occurrence of an already activated inflammatory response in RTT fibroblasts that, on the other hand, seem unable to respond to further pro-inflammatory challenges.

Our results on the constitutively activated status of NF-κB p65 signaling with the subsequent increased levels of IL-1β; production of which is induced only in response to inflammatory *stimuli* [45], may be likely related to the oxinflammation condition typical of RTT [6,38]. Indeed, multiple elements support the implication of a chronic, low-grade inflammation in RTT, including high levels of inflammatory markers and deregulation of acute phase response (APR) proteins, an unbalanced plasma cytokines profile coupled with an abnormal morphology of peripheral blood mononuclear cells (PBMCs) [6]. Moreover, we recently demonstrated an increased gene expression of arachidonate 15-lipoxygenase (ALOX15) in RTT PBMC [44]. This enzyme is able to oxidize polyunsaturated fatty acids such as linoleic acid, producing 13- and 9- hydroxyoctadecadienoic acid (13-HODE and 9-HODE, respectively); levels of these two compounds were found also increased in RTT serum [44]. High HODEs levels are able to exert pro-inflammatory effects such as the induction of cytokines and cell adhesion molecules expression, the modulation of immune cells chemotaxis and monocyte adhesion to vascular endothelial cells and the activation of transcription factors including NF-κB [46]. In addition, beyond the subclinical inflammation, also the redox imbalance, widely observed in RTT, could be another plausible mechanism able to induce the up-regulation of redox-sensitive transcriptional factors such as NF-κB [47]. Of note, an aberrant NF-κB signaling has been already reported in RTT and related to MECP2 deficiency, in both patients and animal models. Alterations of the NF-kB pathway were first revealed in a transcriptional profiling study on the whole blood of RTT patients [48]. Then, the possible involvement of MECP2 in immune function regulation was suggested by the enhanced NF-κB signaling coupled with an increased expression of inflammatory cytokines (*i.e.,* TNFα, IL-6, and IL-3) found in MECP2-deficient human PBMCs and in the human monocyte line THP1 [49,50]. In line with these works, microglia and macrophages from *Mecp2*-deficient mice displayed a deregulated inflammatory response with an abnormal transcriptional expression of inflammatory genes after TNFα stimulation [51]. A study by Kishi and colleagues [52] on the cortex of *Mecp2*-null mice demonstrated an abnormal upregulation of NF-κB signaling, which reduction ameliorated the dendritic complexity of callosal projection neurons and prolonged their normal lifespan. Similarly, a marked decrease in inflammation markers was observed in the cerebellar area of *Mecp2*-knockout mice after treatment with a specific glycogen synthase kinase-3b (Gsk3b) inhibitor that attenuated the nuclear NF-κB activity [53]. Together, these evidence and our results suggest the hypothesis that NF-κB pathway dysregulation could play a significant role in RTT pathophysiology, as already proven in rheumatoid arthritis, inflammatory bowel disease, multiple sclerosis, atherosclerosis, systemic lupus erythematosus, type I diabetes, chronic obstructive pulmonary disease and asthma [54].

In addition to regulate multiple aspects of the immune and inflammatory functions, NF-κB also has a role in regulating the activation of inflammasomes by inducing the transcriptional expression of NLRP3, pro-IL-1β and pro-IL-18 [32,39]. Therefore, based on the evidence of a constitutive activation of NF-κB p65 in RTT fibroblasts, we continued our study by analyzing the main components of NLRP3 signaling pathway. In order to achieve an effective inflammasome-mediated response, the presence of the adaptor protein ASC is crucial, since it can recruit the pro-caspase-1 through a “caspase activation and recruitment domain” (CARD), facilitating caspase-1 dimerization and activation, thus ensuring the formation of a fully functional inflammasome [55]. About ASC, as expected, LPS + ATP increased markedly the levels of ASC in CTR cells, whereas, although exhibiting significantly higher basal levels of ASC, as compared to CTR fibroblasts, RTT cells did not undergo any change upon LPS/ATP treatment. Several papers showed that ASC gene is under the control of a methylation-sensitive promoter [56,57]. In addition, Webb and colleagues [58], by using an IRIDESCENT algorithm analysis, revealed that ASC is sensitive to MECP2 activity and, moreover, transcriptional silencing of ASC gene, associated with the complete methylation of its promoter region, was observed in prostate cancer [59]. Therefore, we hypothesize that the loss of the transcriptional repression activity by a deficient MECP2 could be a mechanism able to explain the increase of ASC levels in RTT fibroblasts [60]. Furthermore, the increased basal levels of ASC in RTT fibroblasts could also be another hypothetic molecular mechanism able to explain the constitutive NF-κB p65 hyperactivation. Indeed, there is evidence that identified ASC as an upstream regulator of NF-κB signaling [61].

Since NLRP3 protein expression in RTT fibroblasts was found to be substantially unchanged after LPS + ATP treatment, we performed a multiple immunofluorescence-based analysis of NLRP3 and ASC, in order to estimate the interaction between these proteins to determine the inflammasome assembly process [20]. Our results provided evidence of an increased interaction between NLRP3 and ASC in unstimulated RTT cells. Furthermore, as expected, control fibroblasts showed increased NLRP3/ASC co-distribution, following the pro-inflammatory challenge, whereas RTT cells were unable to display a similar behavior. Such results are consistent with the data related to NF-κB p65 signaling and cytosolic ASC levels discussed above. The pre-activated status of NLRP3/ASC inflammasome could be also linked to the redox imbalance and the mitochondrial dysfunction, aspects repeatedly described in RTT [42]. Indeed, mitochondrial events have been associated with NLRP3 activation in several different pathological conditions [62]. By acting upstream of the NLRP3 activation, mitochondria can provide ROS to induce NLRP3 oligomerization and be a platform for inflammasome assembly [63,64]. Interestingly, recent findings also revealed a role for HODEs, that we found increased in RTT serum [44], as positive modulators of NLRP3 inflammasome assembly and caspase-1 activation [65].

In addition, the LPS + ATP treatment enhanced the levels of the cleaved form of caspase-1 (p20) in CTR cells, in coherence with the increased co-localization of the sensor NLRP3 and the adaptor ASC proteins within the cytoplasm, thus confirming that the LPS + ATP promoted the functional assembly of inflammasome in fibroblasts from healthy individuals. In basal condition, RTT cells were found to have unchanged levels of CASP1 p20. However, the basal activation state of the CASP1 seemed to be higher in RTT fibroblasts than in CTR cells, as shown by the markedly lower pro-CASP1 protein levels found in fibroblasts from RTT patients. This idea was confirmed by the fact that RTT cells did not undergo any statistically significant change of CASP1 p20 protein level upon pro-inflammatory *stimulus*. In addition, since images on cleaved IL-1β showed an increased green signal in un-treated RTT fibroblasts, similar to the fluorescence intensity of LPS plus ATP stimulated CTR fibroblasts, we can suppose that CASP1 is able to properly work in the cleavage and maturation of IL-1β. While this finding requires further investigations, a plausible explanation for the levels of CASP1 (pro-form and mature form) observed in RTT could be found in an enhanced extracellular release trough extracellular vesicles like exosomes [33,34]. Indeed, in addition to NLRP3 and ASC specks [66,67], also caspase-1 is found in extracellular vesicles. Moreover, recent papers revealed that pro-caspase-1 could be also activated in extracellular compartments, leading to the subsequent cleavage of its substrates in exocytotic secretory pathways, such as exosomes, to disseminate the inflammatory signals to adjacent cells and tissues [68].

Based on the evidence of the extracellular release of inflammasome components and a recent report that showed increased concentrations of caspase-1, IL-1β and IL-18 in serum samples from ASD patients [22] (clinically similar to RTT), we decided to confirm our *in vitro* results on RTT fibroblasts also in the serum from RTT patients. The increased serum levels of ASC oligomers and IL-18 corroborated our cellular findings on the occurrence of a deregulated inflammasome pathway in RTT. Indeed, there is evidence that ASC oligomers remain catalytically active in circulation and, after phagocytosis by adjacent cells, can propagate inflammasome activation, thereby augmenting the pro-inflammatory cascade [67].

Therefore, taken together our data suggest that de-regulated inflammasome activation may have a role in the occurrence of a subclinical inflammatory status in RTT. Several findings demonstrated that a low-grade inflammatory response, avoiding a negative feedback regulation, together with the presence of ROS during the inflammatory processes play a key role in several pathologies: genetic diseases [11,69], diabetes [70], cardiovascular disease [71], cancer [72], and neurodegenerative disorders, such as Alzheimer's and Parkinson's disease [73]. Some of these pathologies have been associated to inflammasome de-regulated activation [21,32].

In addition, several authors demonstrated that ROS production, via the NAD(P)H-oxidase (NOX) activity, induces the formation and activation of NLRP3 inflammasome in different *in vitro* and *in vivo* models [74,75]. Cervellati and co-workers [7] demonstrated that RTT fibroblasts exhibited high NOX enzymatic activity, together with increased levels of superoxide anion and hydrogen peroxide. Hence, the reported inflammasome activation in RTT may be likely due also to a NOX-dependent response.

**Scheme 1:**
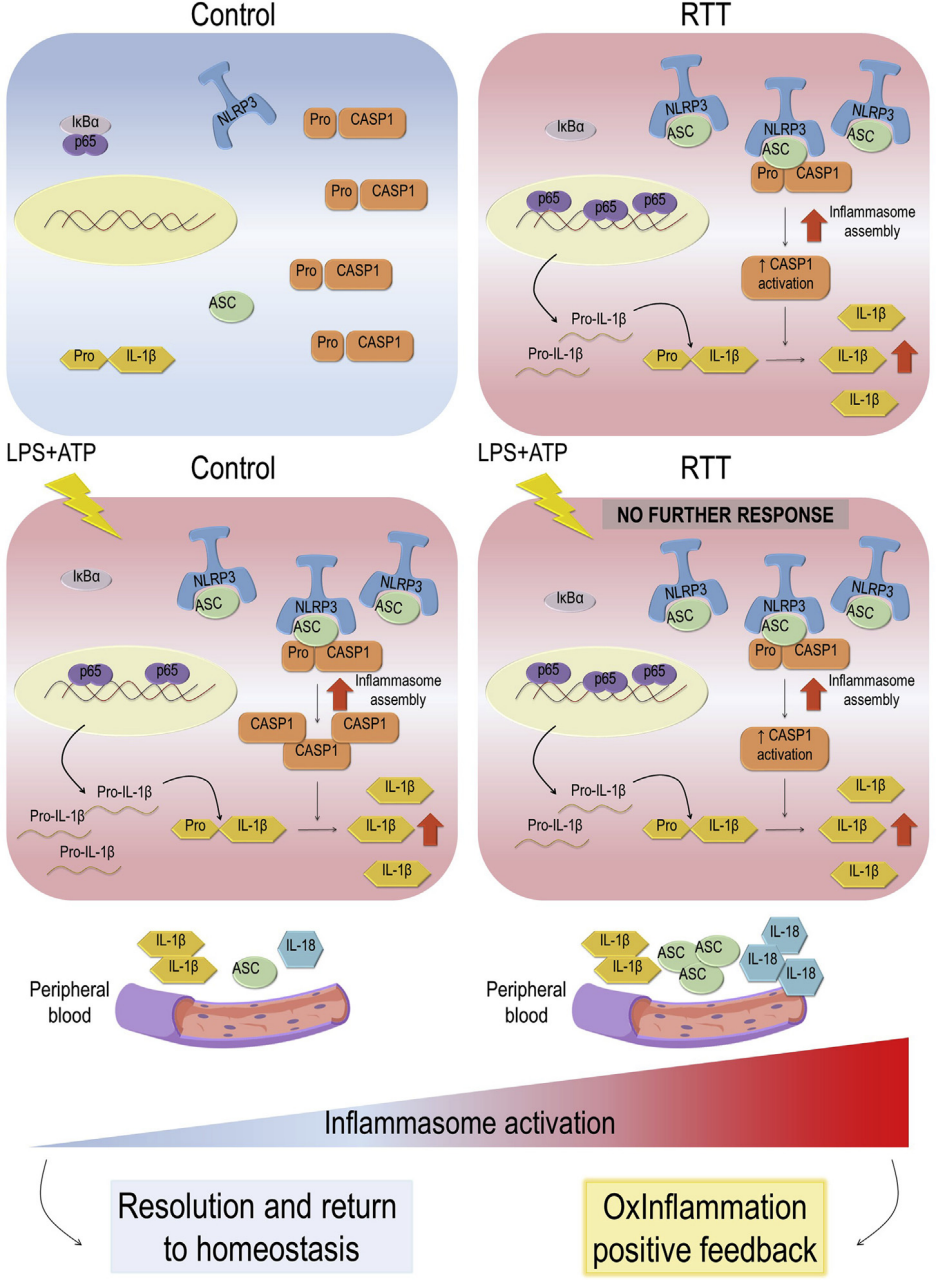
Proposed mechanisms of inflammasome involvement in RTT OxInflammation. The scheme summarizes the basal and LPS + ATP-stimulated status of the inflammasome pathway by comparing the responses of CTR and RTT fibroblasts. As showed in the right panels, RTT fibroblasts were unable to further activate an inflammasome-dependent response to the pro-inflammatory stimulus with LPS and ATP (bottom panel). Indeed, already in basal conditions (upper panel), they exhibited a pre-activated state of the inflammasome machinery, consisting in increased NLRP3/ASC interaction, CASP1 activation and cleaved IL-1β expression, coupled with an enhanced nuclear translocation of NF-κB p65 subunit. In addition, the deregulation of inflammasome pathway was associated with increased levels of inflammasome components in patient-derived serum. In conclusion, a state of constitutive inflammasome activation could play a key role in RTT oxinflammation by fueling and perpetuating the subclinical inflammatory condition observed in this syndrome.

Our experiments show that RTT fibroblasts were unable to activate an inflammasome-dependent response to pro-inflammatory *stimuli*, since they exhibited an already altered machinery, thus suggesting a role of inflammasome in the subclinical inflammatory status, characterizing the pathology (Scheme 1) [6,38]. It is known that stimulation of the immune system in response to sterile insults can lead to chronic debilitating conditions. Hence, for insulted cells it is crucial to coordinate the recognition, initiation, and elaboration of signals inducing inflammasome response, in order to promote the resolution. Whereas a dysregulation of this system can result in disease [14].

## 5 Conclusions

In conclusion, we report that, along with oxidative stress and mitochondrial dysfunction, a state of constitutive activation of the inflammasome system occurs in RTT, which, in turn, may feed and perpetuate the subclinical inflammatory condition observed in this disease.

## Declaration of competing interest

The authors declare no conflict of interests.
