# Supplementary material for: Altered inflammasome machinery as a key player in the perpetuation of Rett syndrome oxinflammation
Source: Redox Biol. 2019 Oct 6;28:101334. doi: 10.1016/j.redox.2019.101334 (PMC6812177; doi:10.1016/j.redox.2019.101334)
Supplement: Multimedia component 1 [file mmc1.ppt]

## Slide 1
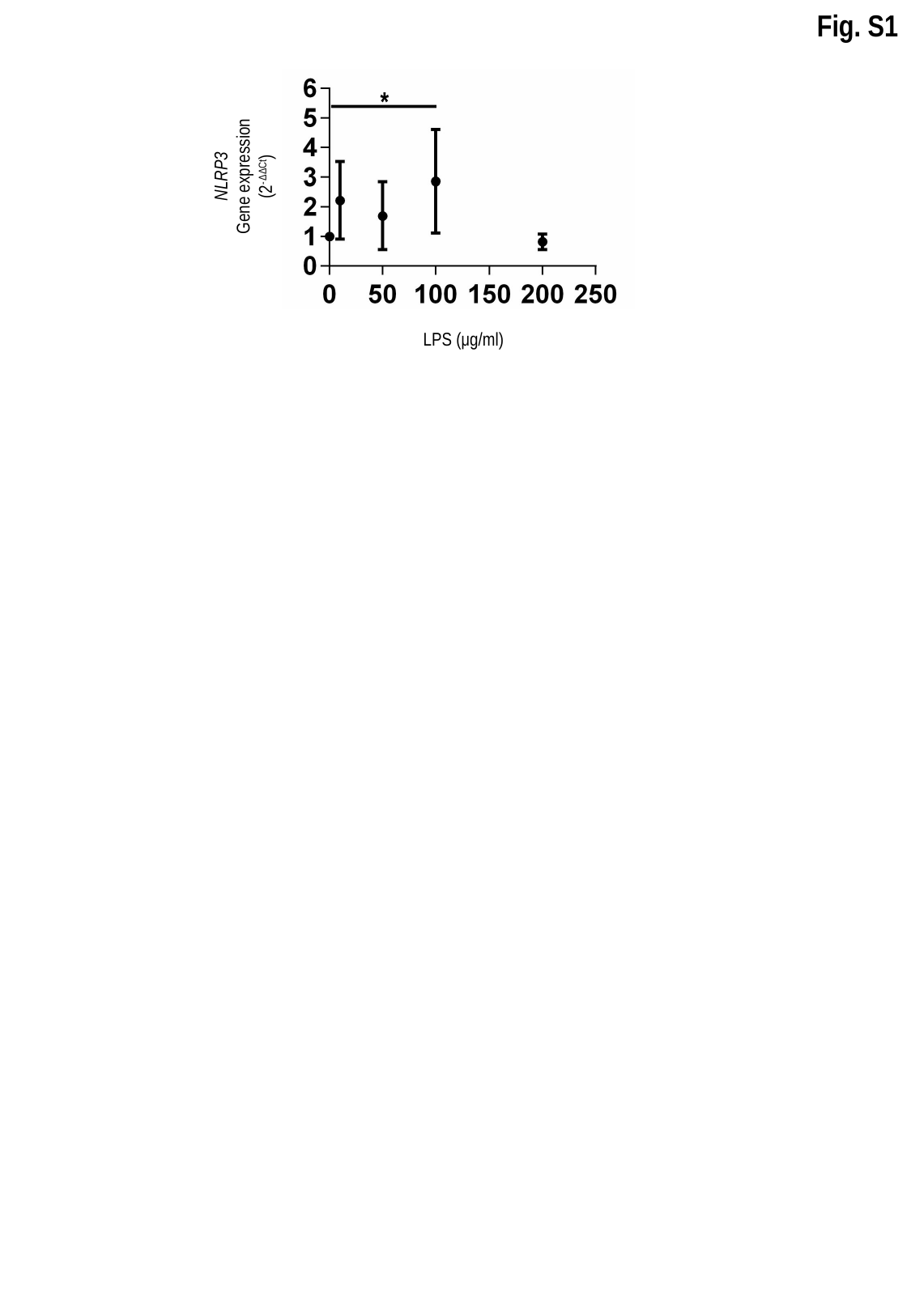

Fig. S1
*
NLRP3
Gene expression
(2- ΔΔCt)
LPS (μg/ml)

## Slide 2
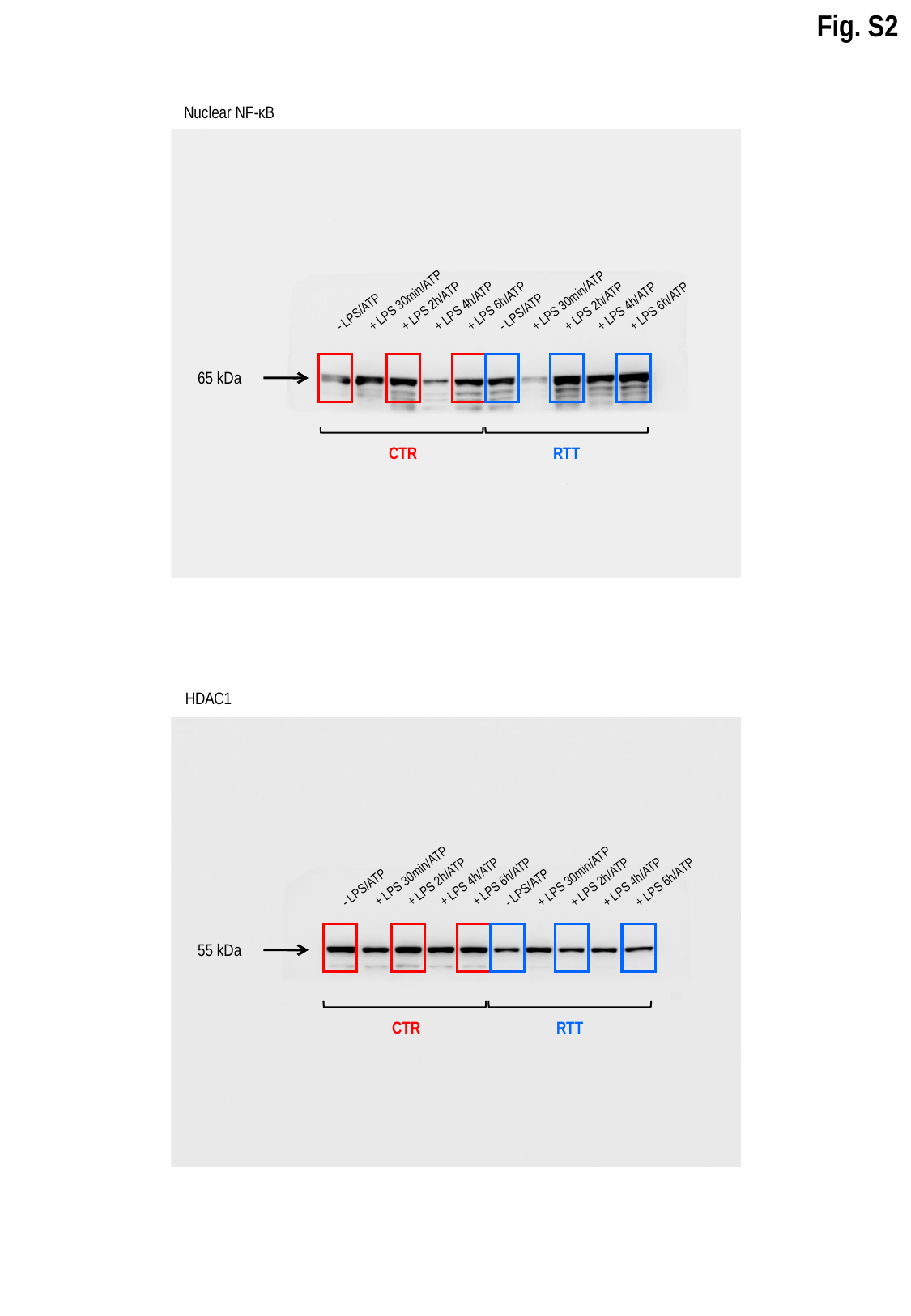

Fig. S2
Nuclear NF-κB
+ LPS 30min/ATP
+ LPS 2h/ATP
+ LPS 4h/ATP
+ LPS 6h/ATP
- LPS/ATP
- LPS/ATP
+ LPS 30min/ATP
+ LPS 2h/ATP
+ LPS 4h/ATP
+ LPS 6h/ATP
65 kDa
CTR
RTT
HDAC1
+ LPS 30min/ATP
+ LPS 2h/ATP
+ LPS 4h/ATP
+ LPS 6h/ATP
- LPS/ATP
- LPS/ATP
+ LPS 30min/ATP
+ LPS 2h/ATP
+ LPS 4h/ATP
+ LPS 6h/ATP
55 kDa
CTR
RTT

## Slide 3
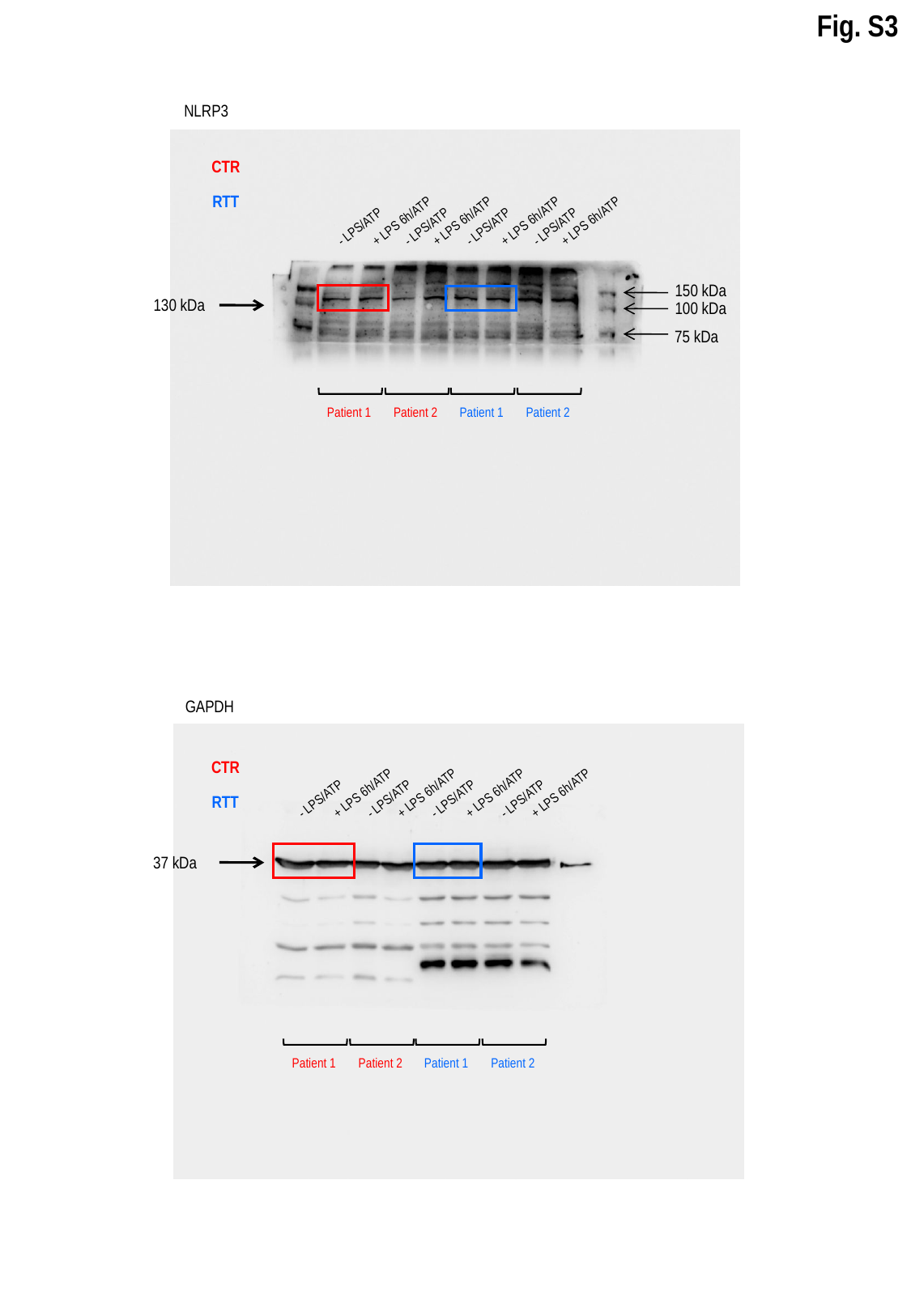

Fig. S3
NLRP3
CTR
- LPS/ATP
+ LPS 6h/ATP
- LPS/ATP
+ LPS 6h/ATP
- LPS/ATP
+ LPS 6h/ATP
- LPS/ATP
+ LPS 6h/ATP
RTT
150 kDa
130 kDa
100 kDa
75 kDa
Patient 1
Patient 2
Patient 1
Patient 2
GAPDH
- LPS/ATP
+ LPS 6h/ATP
- LPS/ATP
+ LPS 6h/ATP
- LPS/ATP
+ LPS 6h/ATP
- LPS/ATP
+ LPS 6h/ATP
CTR
RTT
37 kDa
Patient 1
Patient 2
Patient 1
Patient 2

## Slide 4
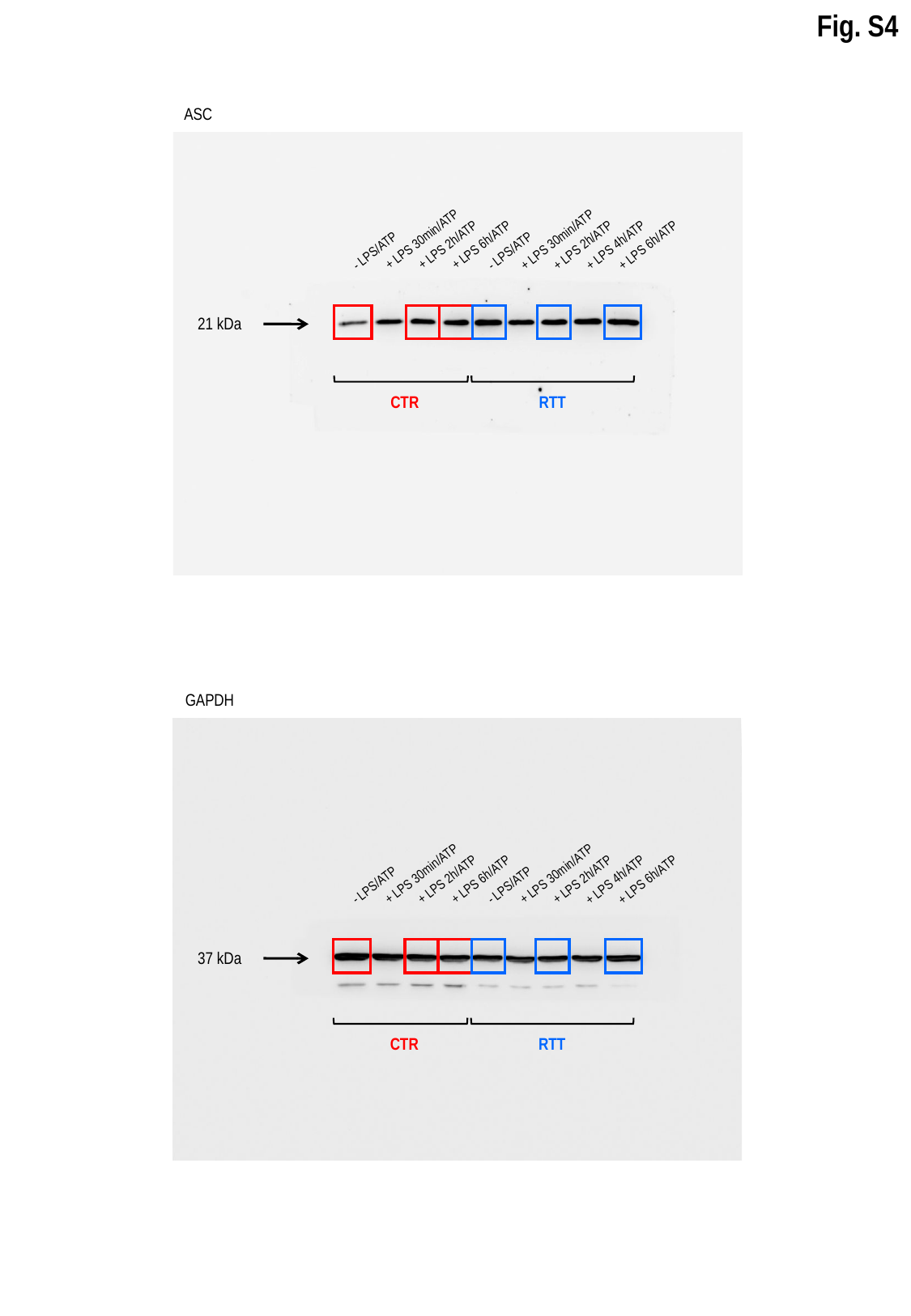

Fig. S4
ASC
+ LPS 30min/ATP
+ LPS 2h/ATP
+ LPS 6h/ATP
- LPS/ATP
- LPS/ATP
+ LPS 30min/ATP
+ LPS 2h/ATP
+ LPS 4h/ATP
+ LPS 6h/ATP
21 kDa
CTR
RTT
GAPDH
+ LPS 30min/ATP
+ LPS 2h/ATP
+ LPS 6h/ATP
- LPS/ATP
- LPS/ATP
+ LPS 30min/ATP
+ LPS 2h/ATP
+ LPS 4h/ATP
+ LPS 6h/ATP
37 kDa
CTR
RTT

## Slide 5
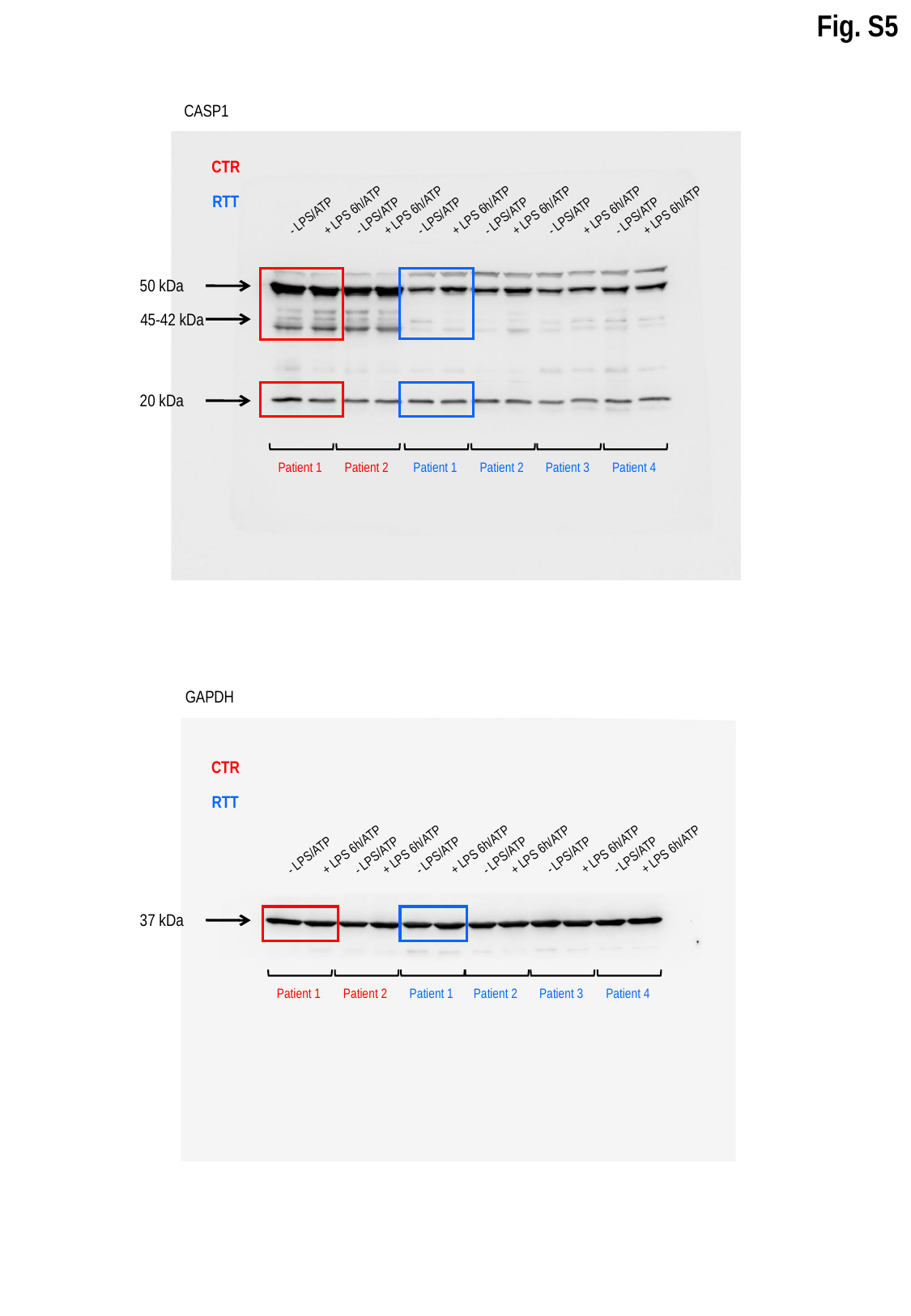

Fig. S5
CASP1
CTR
- LPS/ATP
+ LPS 6h/ATP
- LPS/ATP
+ LPS 6h/ATP
- LPS/ATP
+ LPS 6h/ATP
- LPS/ATP
+ LPS 6h/ATP
- LPS/ATP
+ LPS 6h/ATP
- LPS/ATP
+ LPS 6h/ATP
RTT
50 kDa
45-42 kDa
20 kDa
Patient 1
Patient 2
Patient 1
Patient 2
Patient 3
Patient 4
GAPDH
CTR
RTT
- LPS/ATP
+ LPS 6h/ATP
- LPS/ATP
+ LPS 6h/ATP
- LPS/ATP
+ LPS 6h/ATP
- LPS/ATP
+ LPS 6h/ATP
- LPS/ATP
+ LPS 6h/ATP
- LPS/ATP
+ LPS 6h/ATP
37 kDa
Patient 1
Patient 2
Patient 1
Patient 2
Patient 3
Patient 4

## Slide 6
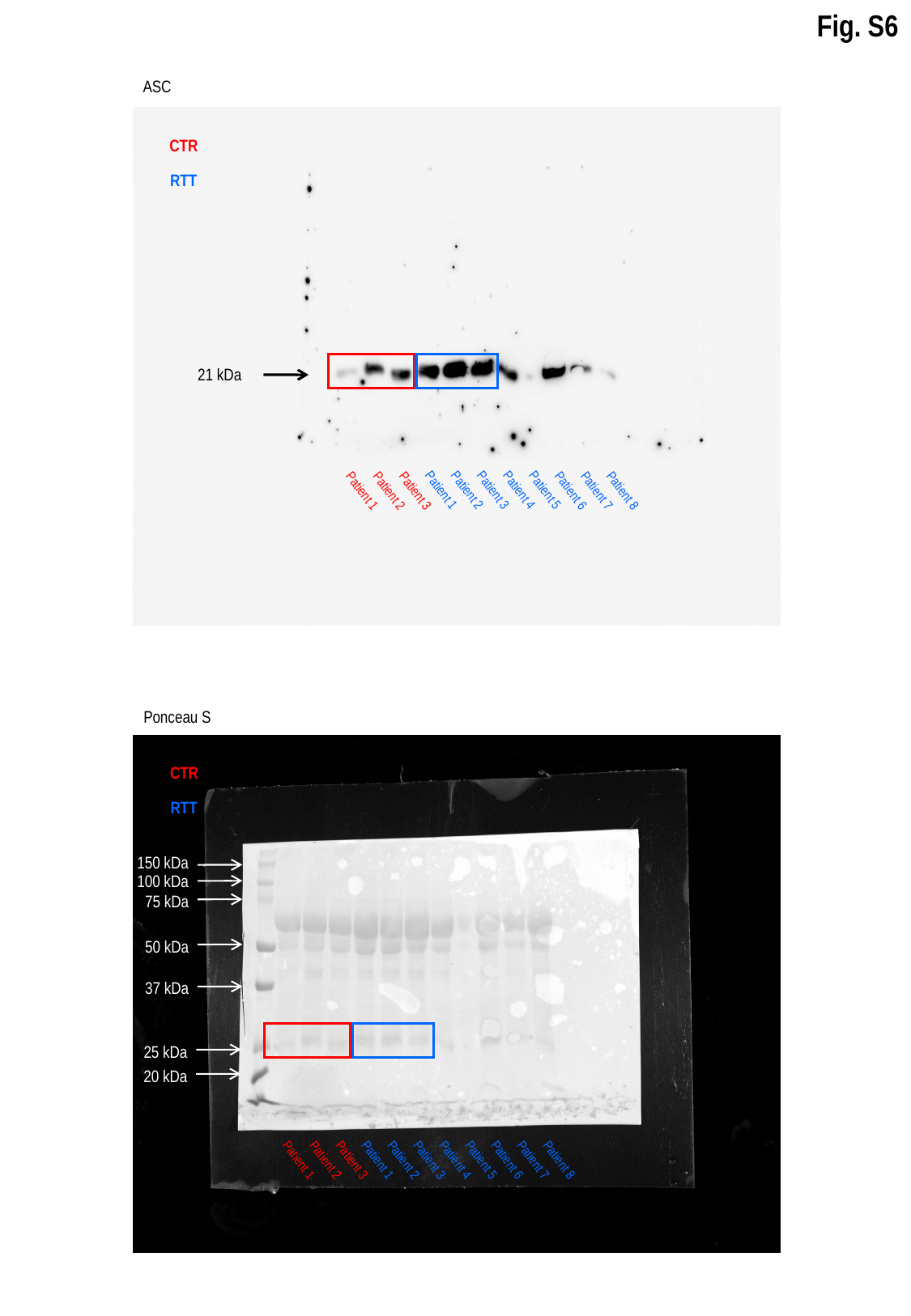

Fig. S6
ASC
CTR
RTT
21 kDa
Patient 1
Patient 2
Patient 3
Patient 4
Patient 5
Patient 6
Patient 7
Patient 8
Patient 2
Patient 1
Patient 3
Ponceau S
CTR
RTT
150 kDa
100 kDa
75 kDa
50 kDa
37 kDa
25 kDa
20 kDa
Patient 4
Patient 6
Patient 8
Patient 3
Patient 5
Patient 7
Patient 1
Patient 2
Patient 1
Patient 3
Patient 2

## Slide 7
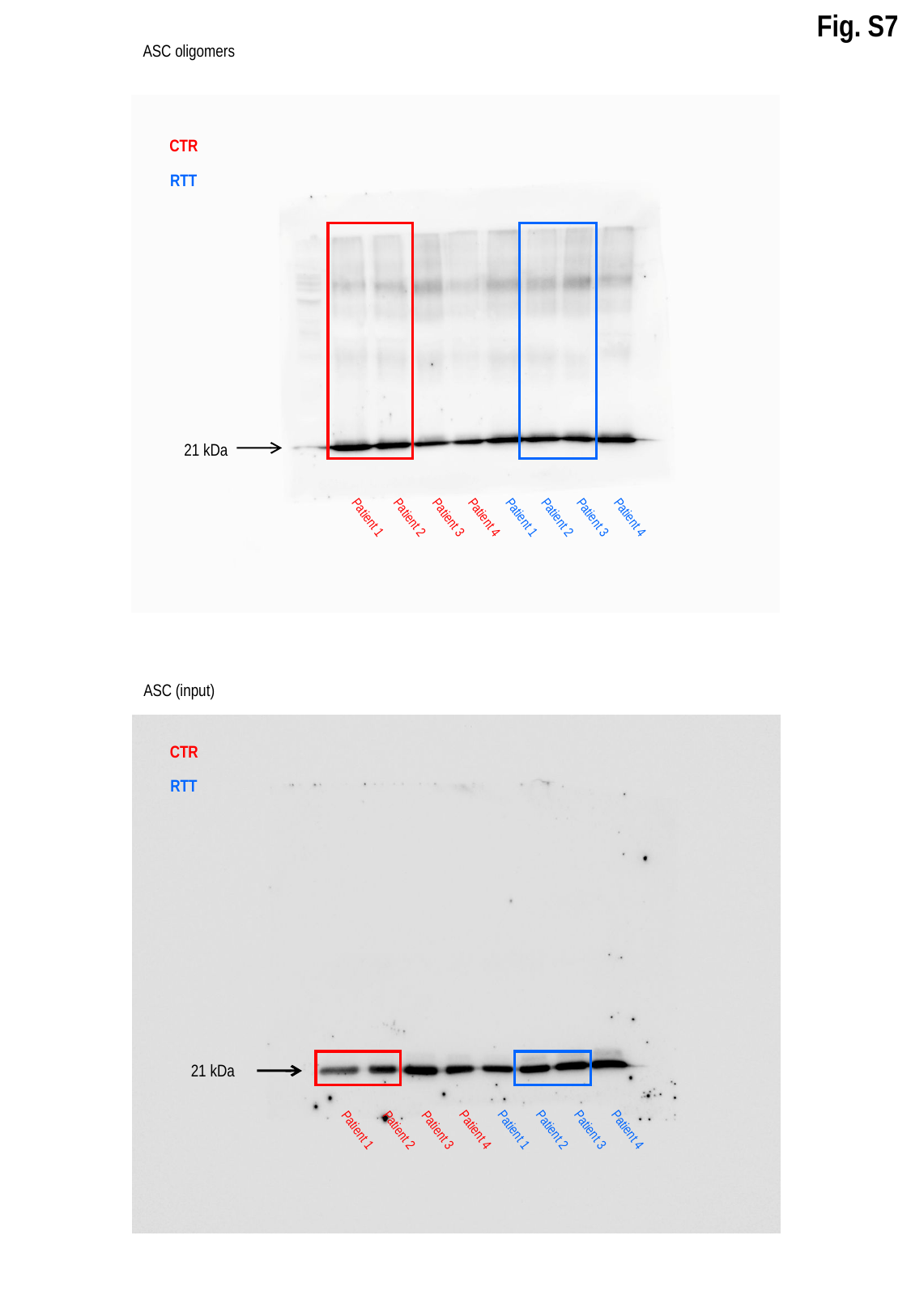

Fig. S7
ASC oligomers
CTR
RTT
21 kDa
Patient 1
Patient 2
Patient 4
Patient 3
Patient 4
Patient 1
Patient 2
Patient 3
ASC (input)
CTR
RTT
21 kDa
Patient 4
Patient 1
Patient 3
Patient 4
Patient 2
Patient 1
Patient 2
Patient 3
