# Supplementary material for: Altered inflammasome machinery as a key player in the perpetuation of Rett syndrome oxinflammation
Source: Redox Biol. 2019 Oct 6;28:101334. doi: 10.1016/j.redox.2019.101334 (PMC6812177; doi:10.1016/j.redox.2019.101334)
Supplement: Multimedia component 3 [file mmc3.docx]

**Supplementary material caption**

**Supplementary Figure S1.** The LPS concentration (100 μg/ml) was chosen based on a significant increase of *NLRP3* gene expression, evaluated through a dose-response curve obtained by incubating cells with LPS concentrations ranging from 0 to 200 μg/ml. Results were analyzed by One-way ANOVA, with Dunnett's multiple comparisons test.

**Supplementary Figure S2.** Representative western blot images for NF-κB p65 and the loading control HDAC1 in nuclear extracts from control and RTT fibroblasts stimulated with LPS 100 μg/ml for 30 min, 2, 4 and 6 hours plus ATP 5 mM for 30 min. The selected time points showed in Fig. 1 are indicated by red and blue boxes for control and RTT, respectively.

**Supplementary Figure S3.** Representative western blot images for NLRP3 and the loading control GAPDH in protein extracts of fibroblasts, from 2 control subjects and 2 RTT patients, incubated without or with LPS 100 μg/ml for 6 hours plus ATP 5 mM for 30 min. The selected time points showed in Fig. 2A are indicated by red and blue boxes for control and RTT, respectively.

**Supplementary Figure S4.** Representative western blot images for ASC and the loading control GAPDH in protein extracts from control and RTT fibroblasts stimulated with LPS 100 μg/ml for 2 and 6 hours plus ATP 5 mM for 30 min. The selected time points showed in Fig. 2B are indicated by red and blue boxes for control and RTT, respectively.

**Supplementary Figure S5.** Representative western blot images for caspase 1 and the loading control GAPDH in protein extracts of fibroblasts, from 2 control subjects and 4 RTT patients, incubated without or with LPS 100 μg/ml for 6 hours plus ATP 5 mM for 30 min. The selected time points showed in Fig. 4A are indicated by red and blue boxes for control and RTT, respectively.

**Supplementary Figure S6.** Representative western blot images for ASC and Ponceau S staining in serum samples from 3 control subjects and 8 RTT patients. The selected bands showed in Fig. 5A are indicated by red and blue boxes for control and RTT, respectively.

**Supplementary Figure S7.** Representative western blot images for ASC oligomers and ASC input in serum samples from 4 control subjects and 4 RTT patients. The selected bands showed in Fig. 5B are indicated by red and blue boxes for control and RTT, respectively.
